# Targeting Mosquitoes through Generation of an Insecticidal RNAi Yeast Strain Using Cas-CLOVER and Super PiggyBac Engineering in *Saccharomyces cerevisiae*

**DOI:** 10.3390/jof9111056

**Published:** 2023-10-27

**Authors:** Corey Brizzee, Keshava Mysore, Teresia M. Njoroge, Seth McConnell, Majidah Hamid-Adiamoh, Akilah T. M. Stewart, J. Tyler Kinder, Jack Crawford, Molly Duman-Scheel

**Affiliations:** 1 Demeetra Ag Bio, 2277 Thunderstick Dr. Suite 300, Lexington, KY 40505, USA; cbrizzee@demeetra.com (C.B.); smcconnell@demeetra.com (S.M.); tkinder@demeetra.com (J.T.K.); 2 Department of Medical and Molecular Genetics, Indiana University School of Medicine, 1234 Notre Dame Ave., South Bend, IN 46617, USA; kmysore@iu.edu (K.M.); tenjorog@iu.edu (T.M.N.); mhamidad@iu.edu (M.H.-A.); akilstew@iu.edu (A.T.M.S.); 3 Eck Institute for Global Health, The University of Notre Dame, Notre Dame, IN 46556, USA; 4 Department of Biological Sciences, The University of Notre Dame, Notre Dame, IN 46556, USA

**Keywords:** *Saccharomyces cerevisiae*, engineering, synthetic biology, mosquitoes, shRNA, *Aedes*, *Anopheles*, *Culex*

## Abstract

The global deployment of RNAi yeast insecticides involves transitioning from the use of laboratory yeast strains to more robust strains that are suitable for scaled fermentation. In this investigation, the RNA-guided Cas-CLOVER system was used in combination with Piggybac transposase to produce robust *Saccharomyces cerevisiae* strains with multiple integrated copies of the *Sh.463* short hairpin RNA (shRNA) insecticide expression cassette. This enabled the constitutive high-level expression of an insecticidal shRNA corresponding to a target sequence that is conserved in mosquito *Shaker* genes, but which is not found in non-target organisms. Top-expressing Cas-CLOVER strains performed well in insecticide trials conducted on *Aedes*, *Culex*, and *Anopheles* larvae and adult mosquitoes, which died following consumption of the yeast. Scaled fermentation facilitated the kilogram-scale production of the yeast, which was subsequently heat-killed and dried. These studies indicate that RNAi yeast insecticide production can be scaled, an advancement that may one day facilitate the global distribution of this new mosquito control intervention.

## 1. Introduction

Mosquito-borne illnesses, which are caused by pathogens spread through the bites of infected mosquitoes, are among the most complex infectious diseases to prevent and control. Although mosquito control is the primary mechanism for preventing these illnesses, which cause ~725,000 deaths annually across the globe [1], a lack of compliance with existing control programs, rising insecticide resistance, and concerns for the negative impacts of pesticides on non-target organisms threaten efforts to combat mosquito-borne diseases [2]. Implementation of the World Health Organization (WHO) global plan for insecticide resistance management [3] is dependent on the successful operational deployment of new insecticides [4]. RNA interference (RNAi) technology, which has been used extensively for laboratory gene characterization studies in a wide variety of insects, is being applied toward development of a new species-specific eco-friendly class of insecticides [5]. In the RNAi pathway, long double-stranded RNA (dsRNA) is cleaved into short 20–25 nucleotide-long small interfering RNAs (siRNAs) that silence genes which have a complementary target sequence [6]. Recent high-throughput screens have identified hundreds of RNAi pesticides, each which silences a mosquito gene that is complementary in sequence and required for mosquito survival [5]. Several of the pesticide target sites that are conserved in human-disease-vector mosquito species, but which are not found in non-target organisms [5,7,8,9,10]. These insecticides kill *Aedes* (dengue, Zika, chikungunya, and yellow fever virus vectors), *Culex* (West Nile and lymphatic filariasis vectors), and *Anopheles* (malaria vectors) mosquitoes, but do not impact the survival of non-target organisms [7,8,9,10].

Recent studies have demonstrated that *Saccharomyces cerevisiae* can be engineered to produce RNAi insecticides and that consumption of the yeast results in mosquito death [5]. In addition to being a versatile bioprocessing platform, *S. cerevisiae* lacks components of the RNAi pathway, enabling this organism to become a cell biofactory and delivery system of shRNA insecticides for mosquito control [11]. One such insecticidal yeast strain [8], Sh.463, expresses a short hairpin RNA (shRNA) with a target sequence that is conserved in mosquito *Shaker (Sh)* genes, which encode voltage-gated potassium channels. Consumption of the yeast, which can be heat-inactivated prior to mosquito feedings, was shown to result in death of *Aedes*, *Anopheles*, and *Culex* mosquito larvae. Although consumption of the yeast results in mosquito death, it is not toxic to non-target organisms that lack the Sh.463 target site, which has not yet been identified in sequenced genomes outside of mosquitoes [8]. The species-specificity of this yeast insecticide suggests that it may represent a new class of eco-friendly insecticides.

Although the first-generation Sh.463 yeast insecticides were useful for proof-of-concept studies [8], the laboratory-based parent yeast strain used to generate the insecticides is not suitable for global yeast insecticide deployment or commercial applications. The growth of these yeast strains is insufficient for scaled insecticide production, presumably due in part to the presence of auxotrophic mutations that necessitate the use of complete media during culturing. Moreover, the expression of *Sh.463* shRNA in the first-generation yeast strain was placed under the control of a galactose-inducible promoter [8]. This is not ideal for scaled fermentation, given that the inclusion of galactose in the fermentation media is required for shRNA expression, which could significantly increase the cost of yeast production. Moreover, the generation of yeast strains bearing multiple copies of the shRNA expression cassette could potentially decrease the amount of yeast that must be applied in insecticidal applications, which is anticipated to be more cost-effective. It was therefore hypothesized that multiple copies of an shRNA expression cassette, in which *Sh.463* shRNA expression is placed under control of a constitutively active promoter, could be successfully integrated into the genome of a robust strain of *S. cerevisiae* using the Cas-CLOVER and piggyBac systems.

Cas-CLOVER, like the original CRISPR/Cas9 system, is an RNA-guided system which maintains the simplicity of the original Cas9 system, but employs the dimeric nuclease Clo051 for genome editing [12]. This dimeric nuclease prevents off-target editing and can generate knock-in strains more efficiently. Moreover, the Cas-CLOVER system seamlessly integrates into existing gRNA design and manufacturing platforms and is, therefore, suitable for a variety of different synthetic applications, including the generation of insecticidal yeast strains [12]. The Cas-CLOVER system can be used for the editing of *Saccharomyces cerevisiae*, one of the most frequently used microorganisms in biotechnology, to enable the production of bulk quantities of biochemicals in yeast. Here, this system was utilized in combination with the Super PiggyBac (sPB) transposase/transposon system. sPB recognizes transposon-specific inverted terminal repeats (ITRs), integrating these ITRs and intervening DNA at TTAA sites within the genome. This facilitates straightforward, consistent transgenesis that is not limited by cargo size, and which could be used to integrate biopesticide cargo, such as shRNA expression cassettes, along with a selectable nutritional marker, enabling increased shRNA expression [12]. Here, the Cas-CLOVER and sPB systems were used to generate yeast strains bearing multiple copies of a high-expression *Sh.463* expression cassette.

## 2. Materials and Methods

### 2.1. Yeast Strain Construction

For generation of auxotrophic mutants, Cas-CLOVER was expressed under control of the *ScRNR2* yeast promoter on a CEN/ARS plasmid marked by kanamycin resistance. The *URA3* gene was first targeted using left URA3 and right URA3 gRNAs (Table S1) expressed under the control of an *SNR52* promoter and using an *SNR52* terminator (Table S1). The guides and Cas-CLOVER were expressed from a single plasmid that was transformed into *S. cerevisiae* FL100 with a homologous donor repair (HDR) fragment that was 200 bp upstream of the start codon and 200 bp downstream of the stop codon of the *URA3* gene. Following PCR confirmation that *URA3* had been deleted, the *LEU2* gene was deleted in a similar manner by expressing Cas-CLOVER under the *ScRNR2* yeast promoter on a CEN/ARS plasmid with hygromycin resistance. The *LEU2* gene was targeted with a left LEU2 and a right LEU2 gRNA (Table S1) expressed by an *SNR52* promoter [13], in combination with an *SNR52* terminator [13], and used to delete the *LEU2* gene. The LEU2 guides and Cas-CLOVER were expressed from a plasmid that was transformed into *S. cerevisiae* FL100 (*ura3∆0*) with an HDR fragment that was 200 bp upstream of the start codon and 200 bp downstream of the stop codon of *LEU2.* Deletion of the *LEU2* gene was confirmed by PCR, and the resultant genotype was *MATa*, *ura3∆0*, *leu2∆0*.

sPB transposase was expressed from a *URA3* selection plasmid with a CEN/ARS origin of replication. A fragment bearing the *LEU2* gene under control of the *LEU2d* promoter was inserted between the piggyBac ITRs. A *Sh.463* [8] (Table S1) expression cassette, in which shRNA (Table 1) expression is regulated by the *GAP* promoter [14] and the *CYC1* terminator [15], was inserted into a multi-cloning site within the piggyBac ITRs and upstream of the *LEU2* selection marker. *S. cerevisiae* was then transformed with a 3 transposon:1 transposase ratio (750 ng:250 ng), as per the instructions of the EZ-yeast Transformation Kit (Zymo Research, Irvine, CA, USA). The desired yeast clones were recovered through selection for growth on SCD-Ura plates, followed by a second round of selection on SCD-Leu plates, and the resulting colonies were transferred to 96-well plates for expansion.

Auxotrophies were restored by amplifying 200 bp upstream or downstream of the *URA3* or *LEU2* genes from the parent *S. cerevisiae* FL100 strain. The product was transformed in 1 µg of the nucleotide sequence encoding the minimal promoter *LEU2d* and the *LEU2* gene as described above, with modifications. Yeast grown on selective media (CM-URA or CM-LEU) were chosen for subsequent qPCR analysis in which relative *Sh.463* expression levels were compared to that of control strain DMT9-51.1, which was engineered to express *Sh.463* using only piggyBac integration. Selective media such as CM-URA or CM-LEU were produced with the following media components: Yeast Nitrogen Base (Sigma Aldrich Y0626, Burlington, MA, USA), Yeast Synthetic Drop-out Mix (US Biological Life Sciences D9540-05, Swampscott, MA, USA), 2% Glucose final (Teknova G9050, Hollister, CA, USA), L-Histidine (Fisher Scientific BP382, Waltham, MA, USA), L-Tryptophan (Fisher Scientific BP395, Waltham, MA, USA), Adenine Sulfate (Alfa Aesar A16964, Ward Hill, MA, USA), and either L-Leucine (Fisher Scientific BP385, Waltham, MA, USA) or Uracil (Alfa Aesar A15570, Ward Hill, MA, USA), respectively.

A *S. cerevisiae* FL100 strain with a resultant genotype of *MATa*, *ura3∆0*, *leu2∆0*, *his3∆0*, and *trp1∆0* was used for the construction of a control yeast strain expressing control shRNA [7] in a similar manner, but with the following variations. sPB was expressed from a *URA3* selection plasmid with a CEN/ARS origin of replication. A fragment bearing the *TRP1* gene under control of the *trp1d* promoter was inserted between the piggyBac ITRs. A control shRNA [8] (Table S1) expression cassette containing the *GAP* promoter [14], control shRNA (Table S1), and the *CYC1* terminator [15] was inserted into a multi-cloning site within the piggyBac ITRs and upstream of the *TRP1* selection marker. *S. cerevisiae* was then transformed as described above. The desired yeast clones were recovered through selection for growth on SCD-Ura plates followed by a second round of selection on SCD-Trp plates, and the resulting colonies were transferred to 96-well plates for expansion. Positive corresponding cultures were then pooled and cultured for 24 h in YPD and made chemically competent as per the instructions of the EZ-Yeast Transformation Kit (Zymo Research, Irvine, CA, USA). The chemically competent cells were then used for the subsequent transformation of an additional control expression cassette fragment as in the previous step; however, the expression cassette was cloned into a multi-cloning site upstream of the *HIS3* gene under control of the *HIS3* promoter between the piggyBac ITRs. After recovery on SCD-Ura plates, the second round of selection was performed on SCD-His plates and the resulting colonies treated as described in the previous expansion step. The final round of control shRNA integration was performed using sPB expressed from a *URA3* selection plasmid with a 2-micron origin of replication. The chemically competent cells were then used for subsequent transformation of an additional control expression cassette fragment, as in the previous step; however, the expression cassette was cloned into a multi-cloning site upstream of the *LEU2* gene under control of the *LEU2* promoter between the piggyBac ITRs. After recovery on SCD-Ura plates, the second round of selection was performed on SCD-Leu plates and verified on SCD-LEU-HIS-TRP plates. The resulting colonies were transferred to a 96-well plate for expansion. The resultant uracil auxotrophy was restored as described previously, and the restored genotype is listed in Table 1.

### 2.2. Analysis of Sh.463 Expression

To assess *Sh.463* expression levels via qRT-PCR, cultures were grown for 72 h in 600 µL of YPD at 30 °C, with shaking at 700 rpm in a 96-deep well plate. Cells were pelleted from 200 µL culture at 1000× *g* for 2 min, and the supernatant was aspirated. RNA was extracted as per the instructions of the YeaStar™ RNA Kit (Zymo Research, Irvine, CA, USA) with the following modifications. A volume of 2.5 µL of Zymolyase (Zymo Research E1004, Irvine, CA, USA) was mixed with 80 µL YR Digestion Buffer to resuspend the cell pellet. The suspension was incubated at 37 °C for 60 min. After addition of YR Lysis Buffer and 1:1 volume of ethanol, approximately 450 µL was added to the Zymo-Spin™ IIICG Column in a collection tube and centrifuged at 13,000× *g* for 30 s. RNA was eluted from the column membrane using 40 µL DNase/RNase-Free Water, and the RNA concentrations were taken via a Biotek Gen5 Synergy H1 microplate reader. The RNA was normalized for subsequent cDNA synthesis.

For cDNA synthesis, 500 ng of RNA was added to each reaction per the instructions of the SuperScript™ IV VILO Master Mix with ezDNase (Thermo Fisher Scientific, Waltham, MA, USA) kit. cDNA was diluted 1:10 using nuclease-free water, and 1 µL was used for qRT-PCR. Real-time quantification was performed using PowerUp™ SYBR™ Green Master Mix for qPCR (Applied Biosystems, Foster City, CA, USA) in conjunction with an Applied Biosystems QuantStudio 6 Pro Real-Time PCR system. The following primer sets were used for relative quantification of transcripts: ALG9-Forward 5′-ATCGTGAAATTGCAGGCAGCTTGG-3′ and ALG9-Reverse 5′-CATGGCAACGGCAGAAGGCAATAA-3′, Sh.463-Forward 5′-TCAAGAGATCGAATGCCTAG-3′ and Sh.463-Reverse 5′-TCCTTCCTTTTCGGTTAGAGC-3′. All PCR reactions were performed in 3–4 replicate wells, and relative quantification results were generated by standardizing reactions to *ALG9* levels and *Sh.463* levels of DMT9-51.1 or DMT9-51.1R #1 using the standard ΔΔCt method [16]. Relative *Sh.463* expression levels were compared to that of strain DMT9-51.1, which was engineered to express *Sh.463* using only piggyBac integration.

### 2.3. Whole-Genome Sequencing (WGS) of Engineered Yeast Strains

WGS was performed by Oxford Nanopore Technology, Oxford, UK, and externally by NovaSeq PE150, Novogene, Sacramento, CA, USA, to determine the genomic integration sites of DMT9-52.2R #3 and DMT9-56.10R #3. To summarize the Nanopore sequencing workflow, yeast gDNA was extracted as per the instructions of the New England Bioloabs (NEB) Monarch HMW gDNA Extraction Kit for Tissue (NEB #T3060S/L, Ipswich, MA). After gDNA was extracted, further size selection was performed to deplete shorter DNA fragments and enrich for longer fragments of DNA, as described in the study of Maghini et al. [17]. Ligation sequencing for size-selected yeast gDNA followed the recommended Flongle Flow Cell (FLO-FLG114) protocol [18] using the sequencing kit SQK-LSK114 (Nanopore, Oxford, UK). All sequencing was performed on a MinION Mk1B through MinKNOW 23.04.6. The set runtime was 20 h with live base calling through internal guppy6.3.9 software in MinKNOW. For DMT9-52.2R #3, two separate runs producing 4.16 GB of data were generated with 51.2 k reads and an estimated N50 of 24.35 kb. Three runs with DMT9-56.10R #3 created 12.38 GB of data with 165.52 k reads and an estimate N50 of 18.69 kb. For DMT9-52.2R #3, 433 out of 51,212 passed reads were mapped directly to the piggyBac transposon reference map using Minimap2.24 (plugin for Geneious Prime 2023.2.1 [19]. For DMT9-56.10R #3, 565 out of 152,595 passed reads were mapped directly to the piggyBac transposon reference map as described above. Short-paired reads were generated by Novogene, and raw sequencing reads were mapped directly to the corresponding piggyBac transposon. For DMT9-52.2R #3, 92,544 of 17,329,572 reads mapped to the piggyBac transposon. For DMT9-56.10R #3, 36,259 of 16,663,100 reads mapped to the piggyBac transposon. From the sequences that mapped to the transposon, the flanking sequences of the piggyBac ITRs were located and searched via BLASTn [20] to identify the genomic integration sites. Sequencing data were deposited at the Sequence Read Archive (SRA) at accession PRJNA1024952. 

### 2.4. Pilot Fermentation Studies of the Engineered Strain

Pilot fermentation studies were performed at the Michigan State University Bioeconomy Institute, Lansing, MI. Two separate pilot fermentations were performed with DMT9-56.10R #3 at the 5 L and 10 L scale using two fermentation media recipes, High Cell Density (HCD) [21] and a proprietary Demeetra Fermentation Media (DFM). Seed rounds to produce the necessary inocula for HCD cultivation were grown for 16 h in 50 mL HCD seed media as described by van Hoek et al. [21], and then 42.5 mL of seed 1 was added to 850 mL HCD seed media; seed 2 was grown for 24 h at 30 °C and 250 rpm. Seed rounds for DFM cultivation were grown in standard yeast extract peptone dextrose (YPD) media at 28 °C and 225 rpm. Fed-batch cultures for the HCD pilot were under the following conditions: 30 °C, pH 5.0, and 2 vvm air. O_2_ was supplied if dissolved oxygen reached below 20%. Fed-batch cultures for the DFM pilot were under the same conditions except for the temperature (28 °C) and vessel volume/minute (1 vvm air). Optical density (OD) and *Sh.463* expression samples were taken throughout the 72 h fermentation period at 24 h intervals beginning at fermentation inoculation (timepoint 0). The cells were pelleted and kept at −80 °C then processed as described previously for *Sh.463* expression. The relative expression was compared to *Sh.463* expression at inoculation.

### 2.5. Mosquito Strains and Rearing

*Aedes albopictus* Gainesville (BEI Resources, NIAID, NIH: MRA-804, provided by Sandra A. Allan), *Aedes aegypti* Liverpool-IB12 (LVP-IB12), *Anopheles gambiae* G3 (BEI Resources, NIAID, NIH: Eggs, MRA-112, provided by Mark Q. Benedict), *Culex quinquefasciatus* JH (provided by the CDC to be distributed by BEI Resources, NIAID, NIH: Eggs, NR-43025), and a local Niles, MI strain of *Culex pipiens* mosquitoes were reared as described [22,23].

### 2.6. Larvicide Studies

Yeast was prepared for insecticide assays as previously described [24], except that it was dried through lyophilization. Larvicide assays were performed as described [25] using either insecticidal or control yeast fed to 20 first instar larvae in 500 mL plastic cups with 50 mL distilled water. Mortality, pupariation, and adult emergence rates were determined. At least six replicate trials were performed, and log transformed data were analyzed using ANOVA with a Tukey post hoc test.

### 2.7. Adulticide Studies

First-generation laboratory yeast was prepared as previously described [24] but lyophilized, then added to a 5% sucrose solution and delivered to adult mosquitoes as previously communicated [9]. A sucrose solution alone and sucrose solution containing control yeast with no known mosquito target [8] served as the controls.

For evaluations of the Cas-Clover second-generation strains characterized herein, the protocol of Mysore et al. [9] was used with the following modifications: For each feeding treatment of 25 mosquitoes, 20 mg of lyophilized yeast (treatment or control) was placed on a 6 cm × 6 cm piece of Westham membrane (Westham LLC, Israel). A volume of 100 µL of Westham matrix (Westham LLC, Israel) was added to the pre-weighed yeast and stirred with a toothpick to create a paste on top of the membrane. A second piece of membrane was placed over the yeast and sealed with a heat sealer to create a sachet. The sachet was delivered to mosquitoes in insectary sugar bait trials. The sachet was set in the bottom of the mosquito cage, and the assay was then conducted as described [9]. Engorged females were assessed for behavioral phenotypes, mortality, and morbidity daily for six days. At least six biological replicate experiments were conducted for each treatment, and data were analyzed using ANOVA. LC_50_ and LC_90_ concentrations were determined by generating dose–response curves through variation of insecticidal yeast concentration (by substitution of control yeast) provided in the sugar bait as described [9]. Four replicate experiments with 25 mosquitoes per control or experimental condition were performed, and data were analyzed using Probit analysis with the SPSS program as described [9].

## 3. Results and Discussion

### 3.1. Production of Robust Yeast Strains with Multiple Copies of the Sh.463 shRNA Construct

#### 3.1.1. Yeast Strain Engineering

**Figure 1:**
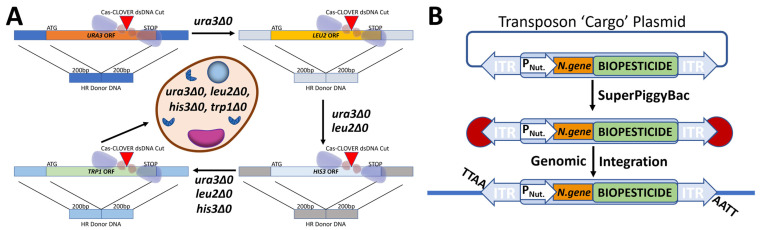
Cas-CLOVER engineering was used to enhance the *S. cerevisiae* bioprocessing platform. (**A**) *S. cerevisiae* was engineered through precise cutting facilitated by use of the dimeric Cas-CLOVER and gRNAs targeting genes, which encode essential amino acids required for growth. (**B**) These engineered *S. cerevisiae* strains can be used in conjunction with the sPB transposase/transposon system to integrate biopesticide cargo along with a selectable nutritional marker (*P_Nut_* = nutritional promoter, *N.gene* = nutrition gene) randomly at ‘TTAA’ sites, allowing for the ease of detection and positive genetic integration.

Production of a yeast strain to be used in conjunction with industrial-sized applications was deemed critical to the future scalability of RNAi yeast-based mosquito control interventions. To address this, Cas-CLOVER was used to create yeast strains bearing multiple auxotrophic gene deletions, including the *ura3* and *leu2* genes (Figure 1A). This yielded a more versatile bioprocessing platform, as these strains are easily rescued by wild-type copies of nutritional marker genes, enabling the selection of transgenics in which the cargo of interest is inserted along with a selectable wild-type copy of the gene. These auxotrophic engineered yeast strains can be used in conjunction with sPB transposase/transposon technology to facilitate the positive selection and efficient detection of the integrated cargo (Figure 1B), including copies of the *Sh.463* expression cassette [8].

**Figure 2:**
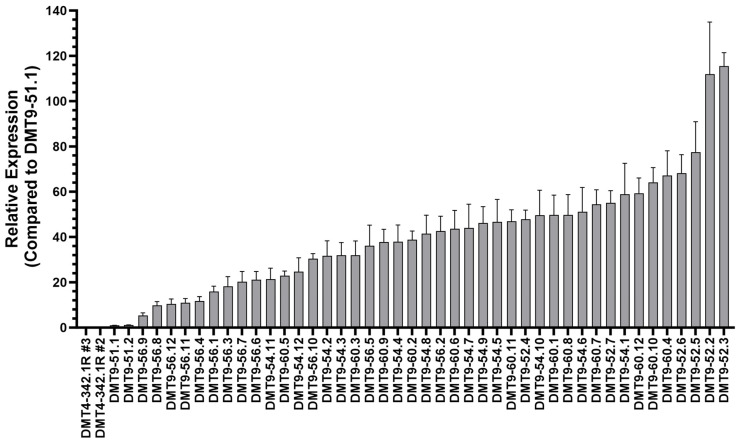
Cas-CLOVER/piggyBac-synthesized yeast strains expressing varying levels of *Sh.463* shRNA. *Sh.463* shRNA expression levels in the indicated yeast strains were assessed through qRT-PCR. The relative expression levels of *Sh.463* shRNA compared to that of the initial piggyBac integration strain DMT9-51.1 are shown. Error bars correspond to SEM.

**Figure 3:**
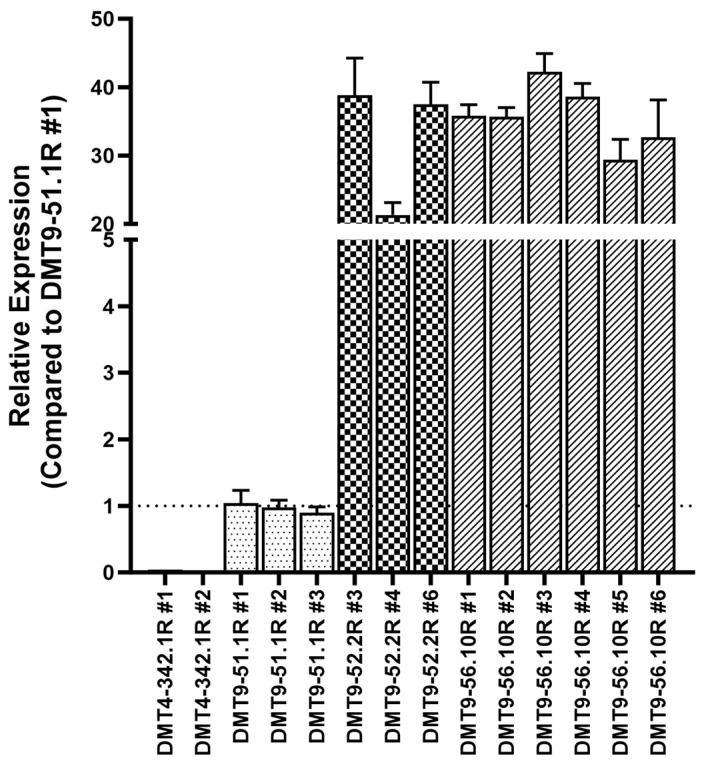
*Sh.463* expression in down-selected yeast strains following auxotrophy restoration. *Sh.463* shRNA expression levels with respect to the DMT4-51.1R #1 are shown. Histogram fill pattern of the restored clones correspond to their ‘parental’ unrestored clone (DMT9-51 Clone #1 (dotted), DMT9-52 Clone #2 (checkered), and DMT9-56 Clone #10 (diagonal lines). RNA levels were assessed via qRT-PCR, and error bars correspond to SEM.

Yeast strains with a range of *Sh.463* expression levels were synthesized (Figure 2). Strains with relatively high levels of *Sh.463* shRNA, which was placed under the control of a constitutive *GAP* promoter [14], were selected for further evaluation. The expression levels of yeast nutritional gene auxotrophies were restored (designated by ‘R’) in these strains (Table 1), and *Sh.463* expression was once again examined (Figure 3). The strains with the highest levels of *Sh.463* shRNA expression, DMT9-52.2R #3 and DMT9-56.10R #3, were selected for more detailed characterization. *Sh.463* levels were ~350–400 times higher than that observed when *Sh.463* was expressed from a pRS426 plasmid [8] that had been transformed into the same yeast strain (Figure 3, see DMT4-342.1R#1, #2, or #3 strains), suggesting that these strains had likely retained multiple copies of the *Sh.463* expression cassette. WGS was utilized to verify the retention of multiple copies of the shRNA expression cassette.

#### 3.1.2. WGS Verifies *Sh.463* Expression Cassette Integration

**Figure 4:**
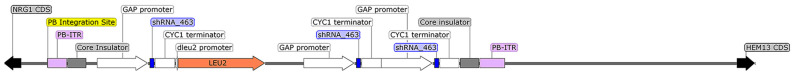
Genomic integration site of the *Sh.463* expression cassette in the DMT9-56.10R #3 strain. A *Sh.463* shRNA expression cassette, bearing three copies of the *Sh.463* expression construct (purple), is stably integrated between the *NRG1* and *HEM13* genes (grey) into position 543,705 on chromosome IV (NC_001136).

**Figure 5:**
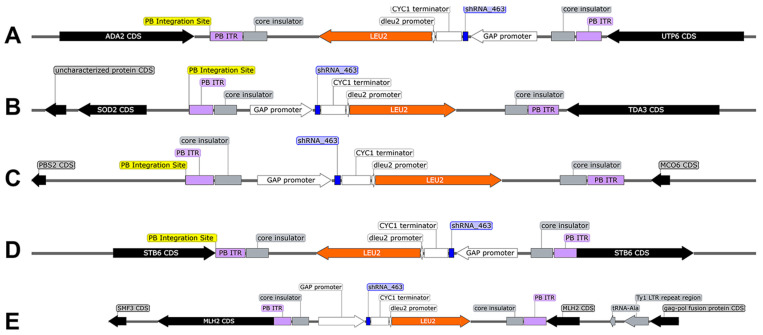
*Sh.463* expression cassette genomic integration sites in the DMT9-52.2R #3 strain. The *Sh.463* shRNA expression cassette is stably integrated into chromosomes IV, VIII, X, XI, and XII. (**A**) The integration sites on chromosome IV (NC_001136; position 1,357,520 between the genes *ADA2* and *UTP6*), (**B**) chromosome VIII (NC_001140; position 124,029 between the genes *SOD2* and *TDA3*), (**C**) chromosome X (NC_001142; position 181,309 between the *PBS2* and *MCO6* genes), (**D**) chromosome XI (NC_001143; position 300,654 intragenic of *STB6*), and (**E**) chromosome XII (NC_001144; position 213,991 intragenic of *MLH2*) are shown.

WGS facilitated the identification of the genomic integration sites of the *Sh.463* expression cassette in DMT9-52.2R #3 and DMT9-56.10R #3 (Table 2). For strain DMT9-56.10R #3, a cassette with three copies of the *Sh.463* expression construct was integrated on chromosome IV (NC_001136) between the genes *NRG1* and *HEM13* (Figure 4). Sequencing of the DMT9-52.2R #3 strain revealed five different genomic integration sites for cassettes bearing single copies of the *Sh.463* expression construct (Figure 5) that had been integrated on chromosomes IV, VIII, X, XI, and XII. Three of the five sites were intergenic, while two of the five genomic integrations were intragenic, occurring in the *STB6* and *MLH2* genes of chromosomes XI and XII, respectively (Table 2 and Figure 5).

The Sh.463 copy number, integration sites, and flanking sequences are indicated for the top yeast strains.

### 3.2. Evaluation of Yeast Larvicidal Activity

**Figure 6:**
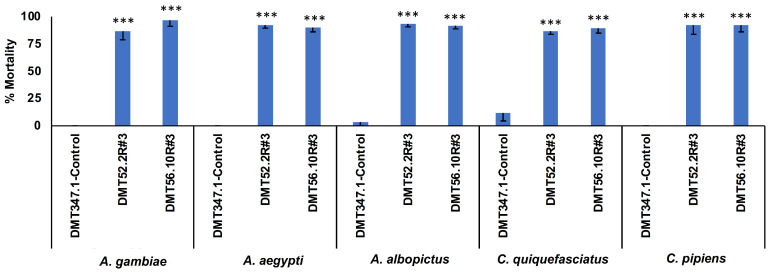
Larvicidal activity of Cas-CLOVER/piggyBac-synthesized yeast strains expressing *Sh.463* shRNA. Larval mortalities following treatments of *A. gambiae*, *A. aegypti*, *A. albopictus*, *C. quinquefasciatus*, and *C. pipiens* larvae with the indicated yeast strains are shown. DMT9-52.2R #3 and DMT9-56.10R #3 yeast treatments induced significant larval mortality with respect to the DMT347.1 control yeast treatments (*** = *p* < 0.001) of each species. A total of 20 larvae were treated with 20 mg of yeast in these assays. Mean mortalities observed in multiple replicate trials are shown, and error bars correspond to SD.

A laboratory yeast strain in which the *Sh.463* expression cassette was integrated into the *S. cerevisiae* genome has been described [8]. The larvicidal activity of this strain was previously confirmed in laboratory and semi-field assays conducted on *A. aegypti* mosquitoes [8]. The larvicidal activity of the yeast was also verified in *A. gambiae* and *C. quinquefasciatus*, in which the target site of the shRNA was conserved [8]. The death of treated mosquito larvae occurred prior to adult emergence in the third or fourth instar of larval development [8]. Based on these results, it was predicted that the second-generation yeast strains would also have larvicidal activity in multiple species of mosquitoes. Yeasts prepared from strains DMT9-52.2R #3 and DMT9-56.10R #3 were evaluated in laboratory larvicide trials conducted on *A. gambiae*, *A. aegypti*, *A. albopictus*, *C. quinquesfasciatus*, and *C. pipiens* larvae. These experiments demonstrated that the dried heat-inactivated DMT9-52.2R #3 and DMT9-56.10R #3 strain yeast, like the first-generation laboratory strains [8], effectively killed mosquito larvae (Figure 6) in the third or fourth instar. Moreover, larval lethality was achieved at dosages that were half (20 mg per 20 larvae) of those used in conjunction with the original first-generation Sh.463 yeast strains (40 mg per 20 larvae; [8]). This is presumably due to the higher expression levels of *Sh.463* observed in the second-generation strains (Figure 3).

### 3.3. Evaluation of Yeast Adulticidal Activity

**Figure 7:**
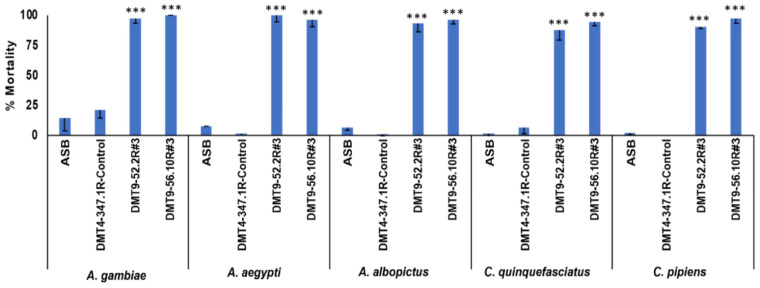
Adulticidal activity of Cas-CLOVER/piggyBac-synthesized yeast strains expressing *Sh.463* shRNA. Adult female morbidities following treatments with the indicated yeast strains are shown in *A. gambiae*, *A. aegypti*, *A. albopictus*, *C. quinquefasciatus*, and *C. pipiens*. DMT9-52.2R #3 and DMT9-56.10R #3 yeast treatments induced significant adult morbidity with respect to DMT4-3471R control-yeast (Control) or sugar-bait-only (ASB) treatments (*** = *p* < 0.001) in each of the indicated species. ~5 µL of 0.2 µg/µL yeast in sugar bait was delivered to each of 25 adult females in six replicate trials conducted with bait station sachets. Mean mortalities observed in multiple replicate trials are shown, and error bars refer to SD.

Recent studies have demonstrated that RNAi yeast can be delivered to mosquitoes as the active ingredient in attractive targeted sugar baits (ATSBs; [9]), an emerging mosquito control technology which capitalizes on the natural sugar-feeding behavior of mosquitoes that are lured to a sugar bait containing an insecticide [26]. A small interfering RNA (siRNA) targeting the Sh.463 target site was previously shown to have adulticidal activity [8], indicating that Sh.463 yeast could potentially be an effective ATSB active ingredient. To evaluate the potential for deploying Sh.463 yeast in this manner, heat-inactivated dried Sh.463 yeast was suspended in sucrose solution and fed to adult female mosquitoes. This first-generation Sh.463 yeast ATSB induced significant adulticidal activity in both *A. aegypti* and *A. gambiae* mosquitoes (*p* < 0.001, Figure S1). Yeast prepared from strains DMT9-52.2R #3 and DMT9-56.10R #3 was then evaluated in laboratory ATSB assays which were conducted using bait station sachets. The adulticidal activity of DMT9-52.2R #3, and DMT9-56.10R #3 yeast was confirmed in adult female *A. gambiae*, *A. aegypti*, *A. albopictus*, *C. quinquefasciatus*, and *C. pipiens* mosquitoes (Figure 7). Both DMT9-52.2R #3, and DMT9-56.10R #3 induced significant adult female mortality with respect to treatments with control yeast or sugar bait alone (ASB) (*p* < 0.001). These data demonstrated that the second-generation Cas-CLOVER strains (Figure 7), like the first-generation Sh.463 strains (Figure S1), could be useful for the control of adult mosquitoes.

As seen in the larvae, adult lethality was observed at dosages that were half (Figure 7, 0.2 µg yeast/µL sugar bait) of those used in conjunction with the original first-generation Sh.463 yeast strains (Figure S1), presumably due to the higher *Sh.463* shRNA levels observed in the second-generation Cas-CLOVER strains (Figure 3). These findings were further verified through the generation of a dose–response curve for strain DMT9-56.10R #3, which corresponded to an LD_50_ of 0.0192 mg/µL (CL 0.0140–0.0298 mg/µL), while that of the original first-generation laboratory strain was 0.199 mg/µL (CL 0.187–0.211 mg/µL), or ~10 fold less. Moreover, despite using half the amount of dried yeast, treatments with the second-generation yeast strains resulted in death within six days, which is also typical for treatments with twice as much of the first-generation RNAi yeast insecticides ([9,10]). These data suggest that use of the second-generation strains could enable the reduction of the amount of yeast deployed in ATSBs, which would be expected to reduce the operational costs of this intervention.

ATSBs are presently being evaluated in an ongoing Phase 3 clinical trial [27] which aims to assess the impact of ATSBs on malaria incidences when the intervention is combined with indoor residual spraying (IRS) and long-lasting insecticidal nets (LLINs) in three different African countries. Dinotefuran, the active ingredient that is being assessed in these trials, is a highly effective insecticide [28], but is much less selective than RNAi-based yeast strains, which were designed to specifically target mosquitoes [5]. The new yeast strains could, therefore, enable the development of second-generation mosquito ATSBs that are more eco-friendly than their predecessors. To this end, the trials conducted herein utilized the same bait and membrane employed in the ongoing ATSB clinical trials [27]. The next step will be to evaluate the RNAi-yeast bait stations in field trials.

### 3.4. Pilot Fermentations

**Figure 8:**
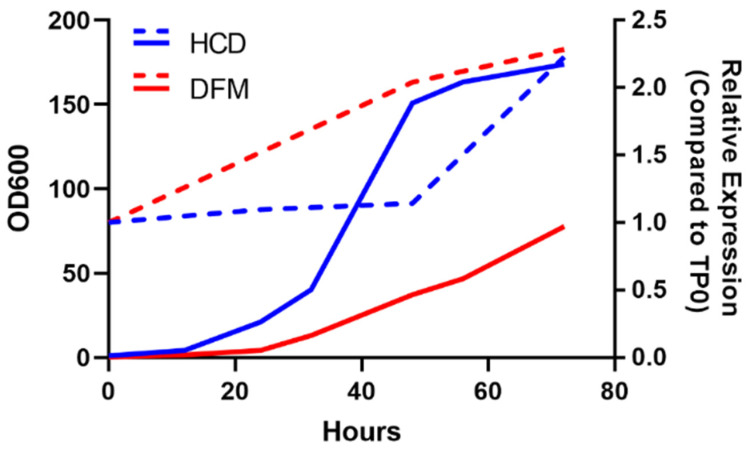
Growth curves and Sh.463 shRNA expression observed in pilot-scaled fermentations with the DMT9-56.10R #3 yeast strain. Growth curves obtained during 72 h pilot-scaled fermentations using two different fermentation medias, High-Cell Density (HCD, blue) and Demeetra’s Fermentation Media (DFM, red), are shown. The relative expression of *Sh.463* shRNA was quantified using qRT-PCR. Dashed lines correspond to OD_600_ readings obtained for cultures grown in each media type: HCD (blue, dashed line) and DFM (red, dashed line).

Following confirmation of the insecticidal activity, pilot fermentations were pursued to assess the feasibility of culturing the DMT9-56.10R #3 yeast strain in industrial-sized fermentations, which would ultimately enable the global deployment of these insecticides. At the 5–10 L fermentation scale, DMT9-56.10R #3 yielded 23.5 g/L and 121.3 g/L dry cell weight (DCW) when cultured using two different sets of fermentation media (Figure 8). The ODs at 600 nm (OD_600_) for cultures prepared with the two different sets of media reached 77.81 (DFM media) and 173.92 (HCD media) over the course of 72 h (Figure 8). In addition to the high growth levels observed, expression of the insecticidal *Sh.463* shRNA remained steady throughout the fermentation irrespective of the media used. The systematic parameters of 5–10 L fermentations are scalable to large-scale fermenters (>100 L), suggesting that the yeast strain will perform similarly well in larger industrial-scaled fermentations.

These efforts to increase yeast production are beneficial, as large-scale field trials to assess the entomological impacts of RNAi adulticide and larvicide deployments will require significantly more yeast than had previously been produced in shake cultures [8]. Such trialing will be required to attain regulatory approvals, for which data supporting the use of the yeast in various different mosquito control capacities will be essential. For example, the second-generation Cas-CLOVER strains will be critical for the pursuit of studies that evaluate the use of larger, slow-release yeast larvicide briquettes for the treatment of large water storage containers, the most productive larval habitats for *Aedes* arboviral mosquitoes [29], as well as for the malaria vector *Anopheles stephensi* [30], a current major threat to urban centers in Asia and Africa.

## 4. Conclusions

In preparation for scaled fermentation, we have generated a robust yeast production strain with multiple integrated shRNA expression cassettes. Insecticidal shRNA expression is driven by a constitutive promoter that enables constant shRNA production at levels ~10 fold higher than the previous laboratory strain. The use of this Cas-CLOVER strain will circumvent the need to use first-generation laboratory strains, eliminating the previous requirement to induce shRNA expression through the addition of galactose to the media, which is not cost-effective at scale. Moreover, the current yeast strain has been rescued of all auxotrophies, bypassing the need to culture it in expensive specialized high-nutrient media containing amino acid supplements. Initial 10 L scaled fermentations with this strain demonstrated that it can be used to produce kilogram-scale quantities of dried yeast without specialized media requirements. These advancements will help scale yeast production for large-scale field trialing and the eventual global distribution of this promising new mosquito control intervention. The technology can also be easily modified to target additional human and agricultural pests.

## 5. Patents

MDS is an inventor on U.S. patent No: 62/361,704/European Application No. 17828458.4, filed by Indiana University. MDS, KM, CB, and JC are co-inventors on a filed patent application with the U.S. Patent Office. Neither of these inventions have affected their interpretation of the data described herein.

## Figures and Tables

**Table 1:** Genotypes of down-selected yeast strains.

shRNA Type	Strain	Genotype	Original Auxotrophy	Restored Genotype
shRNA_463	DMT4-342.1R	*MATa*, *ura3∆0*, *leu2∆0*, *2 um URA3/P_TDH3_-shRNA_463-T_CYC1_*)	Uracil, Leucine	*MATa*, (*2 um URA3/P_TDH3_-shRNA_463-T_CYC1_*)
	DMT9-51.1R	*MATa*, *ura3∆0*, *leu2∆0*, *PiggyBac* (*LEU2/P_TDH3_-shRNA_463-T_CYC1_*), *CEN/ARS* (*URA3/SPBase_Sc-CO*)	Uracil	*MATa*, *PiggyBac* (*LEU2/P_TDH3_-shRNA_463-T_CYC1_*), *CEN/ARS* (*URA3/SPBase_Sc-CO*)
	DMT9-52.2R #3	*MATa*, *ura3∆0*, *leu2∆0*, *PiggyBac* (*leu2d/P_TDH3_-shRNA_463-T_CYC1_*), *CEN/ARS* (*URA3/SPBase_Sc-CO*)	Uracil	*MATa*, *PiggyBac* (*leu2d/P_TDH3_-shRNA_463-T_CYC1_*), *CEN/ARS* (*URA3/SPBase_Sc-CO*)
	DMT9-56.10R #3	*MATa*, *ura3∆0*, *leu2∆0*, *PiggyBac* (*leu2d/P_TDH3_-shRNA_463-T_CYC1_*, *P_TDH3_-shRNA_463-T_CYC1_*, *P_TDH3_-shRNA_463-T_CYC1_*), *CEN/ARS* (*URA3/SPBase-Sc-CO*)	Uracil	*MATa*, *PiggyBac* (*leu2d/P_TDH3_-shRNA_463-T_CYC1_*, *P_TDH3_-shRNA_463-T_CYC1_*, *P_TDH3_-shRNA_463-T_CYC1_*), *CEN/ARS* (*URA3/SPBase-Sc-CO*)
Control	DMT4-347.1R	*MATa*, *ura3∆0*, *leu2∆0*, *his3∆0*, *trp1∆0*, *PiggyBac* (*LEU2/P_TDH3_-shRNA_Ctrl-T_CYC1_*), *2 um* (*URA3/SPBase_Sc-CO*), *PiggyBac* (*HIS3/P_TDH3_-shRNA_Ctrl-T_CYC1_*), *CEN/ARS* (*URA3/SPBase_Sc-CO*) *PiggyBac* (*trp1d/P_TDH3_-shRNA_Ctrl-T_CYC1_*), *CEN/ARS* (*URA3/SPBase_Sc-CO*)	Uracil	*MATa*, *PiggyBac* (*LEU2/P_TDH3_-shRNA_Ctrl-T_CYC1_*), *2 um* (*URA3/SPBase_Sc-CO*), *PiggyBac* (*HIS3/P_TDH3_-shRNA_Ctrl-T_CYC1_*), *CEN/ARS* (*URA3/SPBase_Sc-CO*) *PiggyBac* (*trp1d/P_TDH3_-shRNA_Ctrl-T_CYC1_*), *CEN/ARS* (*URA3/SPBase_Sc-CO*)

For each strain, the type of shRNA produced, original auxotrophy, original and auxotrophy-restored genotypes are shown.

**Table 2:** Summary of WGS data for yeast strains DMT9-56.10R #3 and DMT9-52.2R #3.

Strain	Integration(s)	Sh.463 Copies	Total Copies	Genomic Integration Site	5′ Flanking Sequence (60 bp)	3′ Flanking Sequence (60 bp)
DMT9-56.10R #3	1	3	3	Chromosome IV (543,705)	GAATAACGGAAAAGGAGCCTGCAGCCAGACTGTAGAAAGATGACACTGCCAAGAGAATAA	AAGAAAAAACACCCCAAACACCCTGACCGGCGGCGAAGCCCCTCTGCGCGCTCAACGCGT
DMT9-52.2R #3	5	1	5	Chromosome IV (1,357,520)	ATCGGTTCTTTCCAATTTTTTTTTTTTTTTTTTTTTTTTTTTTTTTTTTTGGTTGTAATA	AAGAATAAACATTTATCTGATATGTAATTGCATTTATAAAATGTACAGTACCGCATTTAA
				Chromosome VIII (124,029)	AAGAAATATATAGATTTAGGTATTCGTTAAATATATACACATTAAATGGCCTCAGAAATT	ATATATAAATAAATAAGCTCTTATATGTACAAATTTGTGCATATACTTTTCTTGACCTTT
				Chromosome X (181,309)	AGTCTAAGCTGAAAGATTATTACTTTCATTTGATTTTTTTATTTTTGAAGCCCCATTTCC	ATCGTTCTCGTGGACGAGATTAAAAATAGAAATGATGTAGAGGAGATGCACTAAACATTG
				Chromosome XI (300,654)	TAACAATTGATAAATTATTTGAAGTATCTTCCAAGACTTCAAACAAAGATATTTTCAAGT	AAAGGTTGTGAAGTCAACTGTTCAAGACATGACTGGCAAAGGAAACTTTATGCATCTATC
				Chromosome XII (213,991)	TTTTTCTTGGCCTCGAAGAAATTTCGAGATACCTTGCTCGTAACCCTCCCAGAAGTTTCC	TATTACTGTAGTCCCCACGGGACAAGATACCTTGTACCTTTTTCCATTGGTAATACCACC

## Data Availability

WGS data that support the findings of this study are openly available at SRA [31] through accession number PRJNA1024952. All the remaining data are available within the text and supplementary information supplied for this article.

## Supplementary Materials

The following supporting information can be downloaded at: https://www.mdpi.com/article/10.3390/jof9111056/s1, Table S1: Nucleic acid sequences. Figure S1: ATSB activity of first-generation Sh.463 yeast strain. Figure S2: Ingestion of Sh.463 Cas-CLOVER/piggyBac-synthesized yeast strains results in adult mosquito mortality.
