# Supplementary material for: Targeting Mosquitoes through Generation of an Insecticidal RNAi Yeast Strain Using Cas-CLOVER and Super PiggyBac Engineering in Saccharomyces cerevisiae
Source: J Fungi (Basel). 2023 Oct 27;9(11):1056. doi: 10.3390/jof9111056 (PMC10672312; doi:10.3390/jof9111056)
Supplement: Supplementary file 1 [file jof-09-01056-s001.zip › jof-2633899-supplementary.pdf]

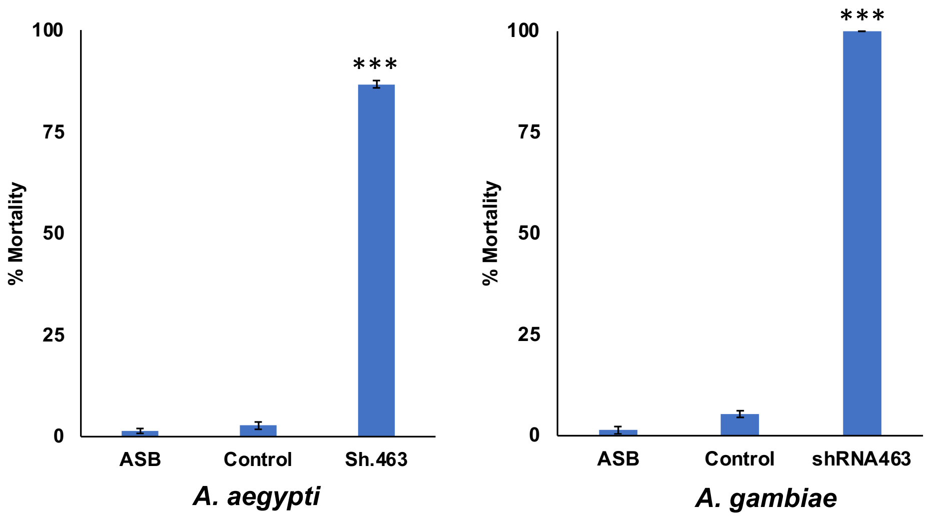

Figure S1. Adulticidal activity of the first-generation laboratory Sh.463 yeast strain. The Sh.463 laboratory strain induced significant adult morbidity in *A. aegypti* (left) and *A. gambiae* (right) when delivered as an ATSB in 5% sucrose. *** = P<0.001 vs 5% sucrose (ASB) or vs. the control yeast treatment (Control). ~5 ul of 0.4 ug/ul yeast in 5% sucrose was delivered to each of 25 adult females in replicate trials. Mean mortalities observed in six replicate trials are shown, and error bars refer to SEM.

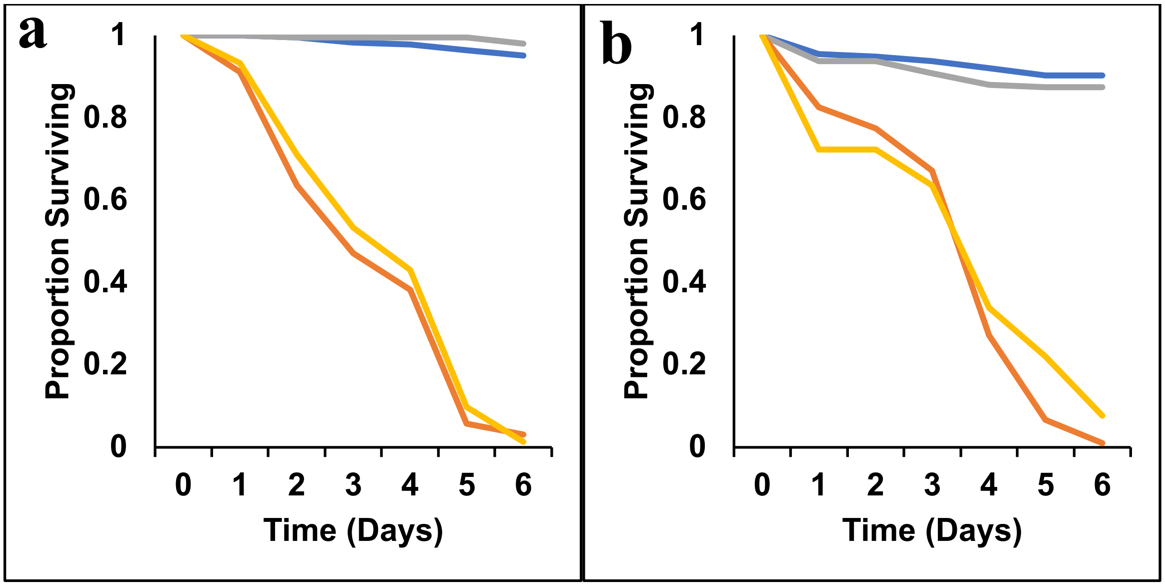

Figure S2. Ingestion of Sh.463 Cas-CLOVER/piggyBac-synthesized yeast strains results in adult mosquito mortality. *A. aegypti* (a) and *A. gambiae* (b) adult females were fed sugar bait alone (blue), or sugar bait with insecticidal DMT9-52.2R#3 yeast (yellow), insecticidal DMT9-56.10R#3 yeast (orange), or control yeast (grey). Although mosquitoes that fed on sugar bait alone or sugar bait with control yeast survived, ingestion of either of the insecticidal yeasts resulted in mosquito death within six days in simulated field trials performed in the insectary. Data were prepared from six replicate trials in which each of 25 adult females fed on ~5 μl of 0.2 μg/μl yeast in sugar bait.

Table S1

**Description**	**Sequence**
shRNA of siRNA.463	gttaaattatctaggcattcgattcaagagatcgaatgcctagataatttaa
Target sequence *Shaker* (AAEL000242)	auuuaaauuaucuaggcauucgaaa
Truncated (defective) *leu2* nutritional promoter	atatatatatttcaaggatataccattcta
3' piggyBac Inverted Terminal Repeat transposable element	ttaaccctagaaagataatcatattgtgacgtacgttaaagataatcatgcgtaaaattgacgcatgtgttttatcggtctgtatatcgaggtttatttattaatttgaatagatattaagttttattatatttacacttacatactaataataaattcaacaaacaatttatttatgtttatttatttattaaaaaaaaacaaaaactcaaaatttcttctataaagtaacaaaactttta
5' piggyBac Inverted Terminal Repeat transposable element	gatatctataacaagaaaatatatatataataagttatcacgtaagtagaacatgaaataacaatataattatcgtatgagttaaatcttaaaagtcacgtaaaagataatcatgcgtcattttgactcacgcggtcgttatagttcaaaatcagtgacacttaccgcattgacaagcacgcctcacgggagctccaagcggcgactgagatgtcctaaatgcacagcgacggattcgcgctatttagaaagagagagcaatatttcaagaatgcatgcgtcaattttacgcagactatctttctagggttaa
piggyBac Core Insulators	gagggacagcccccccccaaagcccccagggatgtaattacgtccctcccccgctagggggcagcagcgagccgcccggggctccgctccggtccggcgctccccccgcatccccgagccggcagcgtgcggggacagcccgggcacggggaaggtggcacgggatcgctttcctctgaacgcttctcgctgctctttgagcctgcagacacctgggggggatacggggaaaa

piggyBac Transposon carrying Sh.463 expression cassette for DMT9-52.2R #3	ttaaccctagaaagataatcatattgtgacgtacgttaaagataatcatgcgtaaaattgacgcatgtgttttatcggtctgtatatcgaggtttatttatta<br>atttgaatagatattaagttttattatatttacacttacatactaataataaattcaacaaacaatttatttatgtttatttatttattaaaaaaaaacaaaaactc<br>aaaatttcttctataaagtaacaaaacttttatagaattctatctagagggacagcccccccccaaagcccccagggatgtaattacgtccctccccc<br>gctagggggcagcagcgagccgcccggggctccgctccggtccggcgctccccccgcatccccgagccggcagcgtgcggggacagccc<br>gggcacggggaaggtggcacgggatcgctttcctctgaacgcttctcgctgctctttgagcctgcagacacctgggggggatacggggaaaaggc<br>ctccaaggccagcttcccacaataagttgggtgaattttggctcattcctcctttctataggattgaggtcagagctttgtgatgggaattctgtggaat<br>gtgtgtcagttagggtgtggaaagtcccgcgatcgctcattatcaatactcgccatttcaaagaatacgtaaataattaatagtagtgattttcctaactt<br>tatttagtcaaaaaattagccttttaattctgctgtaacccgtacatgcccaaaatagggggcgggttacacagaatatataacatcgtaggtgtctgg<br>gtgaacagtttattcctggcatccactaaatataatggagcccgctttttaagctggcatccagaaaaaaaaagaatcccagcaccaaaatattgtttt<br>cttcaccaaccatcagttcataggtccattctcttagcgcaactacagagaacaggggcacaaacaggcaaaaaacgggcacaacctcaatgga<br>gtgatgcaacctgcctggagtaaatgatgacacaaggcaattgacccacgcatgtatctatctcattttcttacaccttctattaccttctgctctctctga<br>tttggaaaaagctgaaaaaaaaggttgaaaccagttccctgaaattattcccctacttgactaataagtatataaagacggtaggtattgattgtaattct<br>gtaaatctatttcttaaacttcttaaattctacttttatagttagtcttttttttagttttaaaacaccagaacttagtttcgacggattctagaactagtggatcc<br>gttaaattatctaggcattcgattcaagagatcgaatgcctagataatttaatttttctcgagtcatgtaattagttatgtcacgcttacattcacgccctc<br>cccccacatccgctctaaccgaaaaggaaggagttagacaacctgaagtctaggtccctatttattttttatagttatgttagtattaagaacgttattta<br>tatttcaaatttttcttttttttctgtacagacgcgtgtacgcatgtaacattatactgaaaaccttgcttgagaaggttttgggacgctcgaaggctttaatt<br>tgccggccggcctgcatatatatatttcaaggatataccattctaatgtctgcccctaagaagatcgtcgttttgccaggtgaccacgttggtcaagaa<br>atcacagccgaagccattaaggttcttaaagctatttctgatgttcgttccaatgtcaagttcgatttcgaaaatcatttaattggtggtgctgctatcgat<br>gctacaggtgttccacttccagatgaggcgctggaagcctccaagaaggctgatgccgttttgttaggtgctgtgggtggtcctaaatggggtacc<br>ggtagtgttagacctgaacaaggtttactaaaaatccgtaaagaacttcaattgtacgccaacttaagaccatgtaactttgcatccgactctcttttag<br>acttatctccaatcaagccacaatttgctaaaggtactgacttcgttgttgtcagagaattagtgggaggtatttactttggtaagagaaaggaagacg<br>atggtgatggtgtcgcttgggatagtgaacaatacaccgttccagaagtgcaaagaatcacaagaatggccgctttcatggccctacaacatgagc<br>caccattgcctatttggtccttggataaagctaatgttttggcctcttcaagattatggagaaaaactgtggaggaaaccatcaagaacgaattcccta<br>cattgaaggttcaacatcaattgattgattctgccgccatgatcctagttaagaacccaacccacctaaatggtattataatcaccagcaacatgtttgg<br>tgatatcatctccgatgaagcctccgttatcccaggttccttgggtttgttgccatctgcgtccttggcctctttgccagacaagaacaccgcatttggtt<br>tgtacgaaccatgccacggttctgctccagatttgccaaagaataaggtcaaccctatcgccactatcttgtctgctgcaatgatgttgaaattgtcatt<br>gaacttgcctgaagaaggtaaggccattgaagatgcagttaaaaaggttttggatgcaggtatcagaactggtgatttaggtggttccaacagtacc<br>accgaagtcggtgatgctgtcgccgaagaagttaagaaaatccttgcttaaaaagattctcttttttttatgatatttgtacataaactttataaatgaaattc<br>ataatagaaacgacacgaaattacaaaatggaatatgttcatagggtagacgaaactatatacgcaatctacatacatttatcaagaaggagaaaaa<br>ggaggatgtaaaggaatacaggtaagcaaattgatactaatggctcaacgtgataaggaaaaagaattgcactttaacattaatattgacaaggag<br>gagggcaccacacaaaaagttaggtgtaacagaaaatcatgaaactatgattcctaatttatatattggaggattttctctaaaaaaaaaaaaaatacaa<br>caaataaaaaacactcaatgacctgaccatttgatggagtttaagtcaataccttccctgcaggtgtcctcacaggaacgaagtccctaaagaaaca<br>gtggcagccaggtttagccccggaattgactggattccttttttagggcccattggtatggctttttccccgtatccccccaggtgtctgcaggctcaa

	agagcagcgagaagcgttcagaggaaagcgatcccgtgccaccttccccgtgcccgggctgtccccgcacgctgccggctcggggatgcggg<br>gggagcgccggaccggagcggagccccgggcggctcgctgctgccccctagcgggggagggacgtaattacatccctgggggctttggggg<br>ggggctgtcccttagcggccgctgatatctataacaagaaaatatatatataataagttatcacgtaagtagaacatgaaataacaatataattatcgta<br>tgagttaaatcttaaaagtcacgtaaaagataatcatgcgtcattttgactcacgcggtcgttatagttcaaaatcagtgacacttaccgcattgacaag<br>cacgcctcacgggagctccaagcggcgactgagatgtcctaaatgcacagcgacggattcgcgctatttagaaagagagagcaatatttcaaga<br>atgcatgcgtcaattttacgcagactatctttctagggttaa
piggyBac Transposon<br><br>carrying Sh.463 expression<br><br>cassette for DMT9-56.10R<br><br>#3	ttaaccctagaaagataatcatattgtgacgtacgttaaagataatcatgcgtaaaattgacgcatgtgttttatcggtctgtatatcgaggtttatttatta<br>atttgaatagatattaagttttattatatttacacttacatactaataataaattcaacaaacaatttatttatgtttatttatttattaaaaaaaaacaaaaactc<br>aaaatttcttctataaagtaacaaaacttttatagaattctatctagagggacagccccccccaaagcccccagggatgtaattacgtccctcccccc<br>gctagggggcagcagcgagccgcccggggctccgctccggtccggcgctccccccgcatccccgagccggcagcgtgcggggacagccc<br>gggcacggggaaggtggcacgggatcgctttcctctgaacgcttctcgctgctctttgagcctgcagacacctgggggggatacggggaaaaggc<br>ctccaaggccagcttcccacaataagttgggtgaattttggctcattcctcctttctataggattgaggtcagagctttgtgatgggaattctgtggaat<br>gtgtgtcagttagggtgtggaaagtcccgcgatcgctcattatcaatactcgccatttcaaagaatacgtaaataattaatagtagtgattttcctaactt<br>tatttagtcaaaaaattagccttttaattctgctgtaacccgtacatgcccaaaatagggggcgggttacacagaatatataacatcgtaggtgtctgg<br>gtgaacagtttattcctggcatccactaaatataatggagcccgctttttaagctggcatccagaaaaaaaaagaatcccagcaccaaaatattgtttt<br>cttcaccaaccatcagttcataggtccattctcttagcgcaactacagagaacaggggcacaaacaggcaaaaaacgggcacaacctcaatgga<br>gtgatgcaacctgcctggagtaaatgatgacacaaggcaattgacccacgcatgtatctatctcattttcttacaccttctattaccttctgctctctctga<br>tttggaaaaagctgaaaaaaaaggttgaaaccagttccctgaaattattcccctacttgactaataagtatataaagacggtaggtattgattgtaattct<br>gtaaatctatttcttaaacttcttaaattctacttttatagttagtcttttttttagttttaaaacaccagaacttagtttcgacggattctagaactagtggatcc<br>gttaaattatctaggcattcgattcaagagatcgaatgcctagataatttaatttttctcgagtcatgtaattagttatgtcacgcttacattcacgccctc<br>cccccacatccgctctaaccgaaaaggaaggagttagacaacctgaagtctaggtccctatttattttttatagttatgttagtattaagaacgttattta<br>tatttcaaatttttcttttttttctgtacagacgcgtgtacgcatgtaacattatactgaaaaccttgcttgagaaggttttgggacgctcgaaggctttaatt<br>tgccggccggcctgcatatatatatttcaaggatataccattctaatgtctgcccctaagaagatcgtcgttttgccaggtgaccacgttggtcaagaa<br>atcacagccgaagccattaaggttcttaaagctatttctgatgttcgttccaatgtcaagttcgatttcgaaaatcatttaattggtggtgctgctatcgat<br>gctacaggtgttccacttccagatgaggcgctggaagcctccaagaaggctgatgccgttttgttaggtgctgtgggtggtcctaaatggggtacc<br>ggtagtgttagacctgaacaaggtttactaaaaatccgtaaagaacttcaattgtacgccaacttaagaccatgtaactttgcatccgactctcttttag<br>acttatctccaatcaagccacaatttgctaaaggtactgacttcgttgttgtcagagaattagtgggaggtatttactttggtaagagaaaggaagacg<br>atggtgatggtgtcgcttgggatagtgaacaatacaccgttccagaagtgcaaagaatcacaagaatggccgctttcatggccctacaacatgagc<br>caccattgcctatttggtccttggataaagctaatgttttggcctcttcaagattatggagaaaaactgtggaggaaaccatcaagaacgaattcccta<br>cattgaaggttcaacatcaattgattgattctgccgccatgatcctagttaagaacccaacccacctaaatggtattataatcaccagcaacatgtttgg<br>tgatatcatctccgatgaagcctccgttatcccaggttccttgggtttgttgccatctgcgtccttggcctctttgccagacaagaacaccgcatttggtt<br>tgtacgaaccatgccacggttctgctccagatttgccaaagaataaggtcaaccctatcgccactatcttgtctgctgcaatgatgttgaaattgtcatt

	gaacttgcctgaagaaggtaaggccattgaagatgcagttaaaaaggttttggatgcaggtatcagaactggtgatttaggtggttccaacagtacc<br>accgaagtcggtgatgctgtcgccgaagaagttaagaaaatccttgcttaaaaagattctcttttttttatgatatttgtacataaactttataaatgaaattc<br>ataatagaaacgacacgaaattacaaaatggaatatgttcatagggtagacgaaactatatacgcaatctacatacatttatcaagaaggagaaaaa<br>ggaggatgtaaaggaatacaggtaagcaaattgatactaatggctcaacgtgataaggaaaaagaattgcactttaacattaatattgacaaggag<br>gagggcaccacacaaaaagttaggtgtaacagaaaatcatgaaactatgattcctaatttatatattggaggattttctctaaaaaaaaaaaaaatacaa<br>caaataaaaaacactcaatgacctgaccatttgatggagtttaagtcaataccttccctgcaggtgtcctcacaggaacgaagtccctaaagaaaca<br>gtggcagccaggtttagccccggaattgactggattcctttttagtcattatcaatactcgccatttcaaagaatacgtaaataattaatagtagtgattt<br>tcctaactttatttagtcaaaaaattagccttttaattctgctgtaacccgtacatgcccaaaatagggggcgggttacacagaatatataacatcgtag<br>gtgtctgggtgaacagtttattcctggcatccactaaatataatggagcccgctttttaagctggcatccagaaaaaaaaagaatcccagcaccaaa<br>atattgttttcttcaccaaccatcagttcataggtccattctcttagcgcaactacagagaacaggggcacaaacaggcaaaaaacgggcacaacct<br>caatggagtgatgcaacctgcctggagtaaatgatgacacaaggcaattgacccacgcatgtatctatctcattttcttacaccttctattaccttctgct<br>ctctctgatttggaaaaagctgaaaaaaaaggttgaaaccagttccctgaaattattcccctacttgactaataagtatataaagacggtaggtattgat<br>tgtaattctgtaaatctatttcttaaacttcttaaattctacttttatagttagtctttttttttagttttaaaacaccagaacttagtttcgacggattctagaacta<br>gtggatccgttaaattatctaggcattcgattcaagagatcgaatgcctagataatttaattttttctcgagtcatgtaattagttatgtcacgcttacattca<br>cgccctccccccacatccgctctaaccgaaaaggaaggagttagacaacctgaagtctaggtccctatttattttttttatagttatgttagtattaagaa<br>cgttatttatatttcaaatttttcttttttttctgtacagacgcgtgtacgcatgtaacattatactgaaaaccttgcttgagaaggttttgggacgctcgaag<br>gctttaatttgcgggtcattatcaatactcgccatttcaaagaatacgtaaataattaatagtagtgattttcctaactttatttagtcaaaaaattagcctttt<br>aattctgctgtaacccgtacatgcccaaaatagggggcgggttacacagaatatataacatcgtaggtgtctgggtgaacagtttattcctggcatcc<br>actaaatataatggagcccgctttttaagctggcatccagaaaaaaaaagaatcccagcaccaaaatattgttttcttcaccaaccatcagttcatagg<br>tccattctcttagcgcaactacagagaacaggggcacaaacaggcaaaaaacgggcacaacctcaatggagtgatgcaacctgcctggagtaaa<br>tgatgacacaaggcaattgacccacgcatgtatctatctcattttcttacaccttctattaccttctgctctctctgatttggaaaaagctgaaaaaaaag<br>gttgaaaccagttccctgaaattattcccctacttgactaataagtatataaagacggtaggtattgattgtaattctgtaaatctatttcttaaacttcttaa<br>attctacttttatagttagtctttttttttagttttaaaacaccagaacttagtttcgacggattctagaactagtggatccgttaaattatctaggcattcgattc<br>aagagatcgaatgcctagataatttaattttttctcgagtcatgtaattagttatgtcacgcttacattcacgccctccccccacatccgctctaaccgaa<br>aaggaaggagttagacaacctgaagtctaggtccctatttattttttttatagttatgttagtattaagaacgttatttatatttcaaatttttcttttttttctgtac<br>agacgcgtgtacgcatgtaacattatactgaaaaccttgcttgagaaggttttgggacgctcgaaggctttaatttgcgggcccattggtatggcttttt<br>ccccgtatcccccccaggtgtctgcaggctcaaagagcagcgagaagcgttcagaggaaagcgatcccgtgccaccttccccgtgcccgggctg<br>tccccgcacgctgccggctcggggatgcggggggagcgccggaccggagcggagccccgggcggctcgctgctgccccctagcgggga<br>gggacgtaattacatccctgggggctttgggggggggctgtcccttagcggccgctgatatctataacaagaaaatatatatataataagttatcacg<br>taagtagaacatgaaataacaatataattatcgtatgagttaaatcttaaaagtcacgtaaaagataatcatgcgtcattttgactcacgcggtcgttata<br>gttcaaaatcagtgacacttaccgcattgacaagcacgcctcacgggagctccaagcggcgactgagatgtcctaaatgcacagcgacggattcg<br>cgctatttagaaagagagagcaatatttcaagaatgcatgcgtcaattttacgcagactatctttctagggttaa

pRS426_463 plasmid	gacgaaagggcctcgtgatacgcctatttttataggttaatgtcatgataataatggtttcttagtatgatccaatatcaaaggaaatgatagcattgaa<br>ggatgagactaatccaattgaggagtggcagcatatagaacagctaaagggtagtgctgaaggaagcatacgataccccgcatggaatgggata<br>atatcacaggaggtactagactacctttcatcctacataaatagacgcatataagtacgcatttaagcataaacacgcactatgccgttcttctcatgta<br>tatatatatacaggcaacacgcagatataggtgcgacgtgaacagtgagctgtatgtgcgcagctcgcgttgcattttcggaagcgctcgttttcgg<br>aaacgctttgaagttcctattccgaagttcctattctctagaaagtataggaacttcagagcgcttttgaaaaccaaaagcgctctgaagacgcacttt<br>caaaaaaccaaaaacgcaccggactgtaacgagctactaaaatattgcgaataccgcttccacaaacattgctcaaaagtatctctttgctatatct<br>ctgtgctatatccctatataacctacccatccacctttcgctccttgaacttgcatctaaactcgacctctacattttttatgtttatctctagtattactcttta<br>gacaaaaaaattgtagtaagaactattcatagagtgaatcgaaaacaatacgaaaatgtaaacatttcctatacgtagtatatagagacaaaatagaa<br>gaaaccgttcataattttctgaccaatgaagaatcatcaacgctatcactttctgttcacaaagtatgcgcaatccacatcggtatagaatataatcggg<br>gatgcctttatcttgaaaaaatgcacccgcagcttcgctagtaatcagtaaacgcgggaagtggagtcaggctttttttatggaagagaaaatagaca<br>ccaaagtagccttcttctaaccttaacggacctacagtgcaaaaagttatcaagagactgcattatagagcgcacaaaggagaaaaaaagtaatcta<br>agatgctttgttagaaaaatagcgctctcgggatgcatttttgtagaacaaaaaagaagtatagattctttgttggtaaaatagcgctctcgcgttgcatt<br>tctgttctgtaaaaatgcagctcagattctttgtttgaaaaattagcgctctcgcgttgcatttttgttttacaaaaatgaagcacagattcttcgttggtaa<br>aatagcgctttcgcgttgcatttctgttctgtaaaaatgcagctcagattctttgtttgaaaaattagcgctctcgcgttgcatttttgttctacaaaatgaa<br>gcacagatgcttcgttcaggtggcacttttcggggaaatgtgcgcggaacccctatttgtttatttttctaaatacattcaaatatgtatccgctcatgag<br>acaataaccctgataaatgcttcaataatattgaaaaaggaagagtatgagtattcaacatttccgtgtcgcccttattcccttttttgcggcattttgcctt<br>cctgtttttgctcacccagaaacgctggtgaaagtaaaagatgctgaagatcagttgggtgcacgagtgggttacatcgaactggatctcaacagc<br>ggtaagatccttgagagttttcgccccgaagaacgttttccaatgatgagcacttttaaagttctgctatgtggcgcggtattatcccgtattgacgccg<br>ggcaagagcaactcggtcgccgcatacactattctcagaatgacttggttgagtactcaccagtcacagaaaagcatcttacggatggcatgacag<br>taagagaattatgcagtgctgccataaccatgagtgataacactgcggccaacttacttctgacaacgatcggaggaccgaaggagctaaccgctt<br>ttttgcacaacatgggggatcatgtaactcgccttgatcgttgggaaccggagctgaatgaagccataccaaacgacgagcgtgacaccacgatg<br>cctgtagcaatggcaacaacgttgcgcaaactattaactggcgaactacttactctagcttcccggcaacaattaatagactggatggaggcggata<br>aagttgcaggaccacttctgcgctcggcccttccggctggctggtttattgctgataaatctggagccggtgagcgtgggtctcgcggtatcattgc<br>agcactggggccagatggtaagccctcccgtatcgtagttatctacacgacggggagtcaggcaactatggatgaacgaaatagacagatcgct<br>gagataggtgcctcactgattaagcattggtaactgtcagaccaagtttactcatatatactttagattgatttaaaacttcatttttaatttaaaaggatct<br>aggtgaagatcctttttgataatctcatgaccaaaatcccttaacgtgagttttcgttccactgagcgtcagaccccgtagaaaagatcaaaggatctt<br>cttgagatcctttttttctgcgcgtaatctgctgcttgcaaacaaaaaaaccaccgctaccagcggtggtttgtttgccggatcaagagctaccaactct<br>ttttccgaaggtaactggcttcagcagagcgcagataccaaatactgtccttctagtgtagccgtagttaggccaccacttcaagaactctgtagcac<br>cgcctacatacctcgctctgctaatcctgttaccagtggctgctgccagtggcgataagtcgtgtcttaccgggttggactcaagacgatagttaccg<br>gataaggcgcagcggtcgggctgaacggggggttcgtgcacacagcccagcttggagcgaacgacctacaccgaactgagatacctacagcg<br>tgagctatgagaaagcgccacgcttcccgaagggagaaaggcggacaggtatccggtaagcggcagggtcggaacaggagagcgcacgag<br>ggagcttccagggggaaacgcctggtatctttatagtcctgtcgggtttcgccacctctgacttgagcgtcgatttttgtgatgctcgtcaggggggc<br>ggagcctatggaaaaacgccagcaacgcggcctttttacggttcctggccttttgctggccttttgctcacatgttctttcctgcgttatcccctgattct

	gtggataaccgtattaccgcctttgagtgagctgataccgctcgccgcagccgaacgaccgagcgcagcgagtcagtgagcgaggaagcggaa<br>gagcgcccaatacgcaaaccgcctctccccgcgcgttggccgattcattaatgcagctggcacgacaggtttcccgactggaaagcgggcagtg<br>agcgcaacgcaattaatgtgagttacctcactcattaggcaccccaggctttacactttatgcttccggctcctatgttgtgtggaattgtgagcggata<br>acaatttcacacaggaaacagctatgaccatgattacgccaagcgcgcaattaaccctcactaaagggaacaaaagctggagctcagtttatcatta<br>tcaatactcgccatttcaaagaatacgtaaataattaatagtagtgattttcctaactttatttagtcaaaaaattagccttttaattctgctgtaacccgtac<br>atgcccaaaatagggggcgggttacacagaatatataacatcgtaggtgtctgggtgaacagtttattcctggcatccactaaatataatggagccc<br>gctttttaagctggcatccagaaaaaaaaagaatcccagcaccaaaatattgttttcttcaccaaccatcagttcataggtccattctcttagcgcaact<br>acagagaacaggggcacaaacaggcaaaaaacgggcacaacctcaatggagtgatgcaacctgcctggagtaaatgatgacacaaggcaatt<br>gacccacgcatgtatctatctcattttcttacaccttctattaccttctgctctctctgatttggaaaaagctgaaaaaaaaaggttgaaaccagttccctga<br>aattattcccctacttgactaataagtatataaagacggtaggtattgattgtaattctgtaaatctatttcttaaacttcttaaattctacttttatagttagtct<br>ttttttagttttaaaacaccagaacttagtttcgacggattctagaactagtggatccgttaaattatctaggcattcgattcaagagatcgaatgcctag<br>ataatttaatttttctcgagtcatgtaattagttatgtcacgcttacattcacgccctccccccacatccgctctaaccgaaaaggaaggagttagacaa<br>cctgaagtctaggtccctatttatttttttatagttatgttagtattaagaacgttatttatatttcaaatttttcttttttttctgtacagacgcgtgtacgcatgta<br>acattatactgaaaaccttgcttgagaaggttttgggacgctcgaaggctttaatttgcggccggtacccaattcgccctatagtgagtcgtattacgc<br>gcgctcactggccgtcgttttacaacgtcgtgactgggaaaaccctggcgttacccaacttaatcgccttgcagcacatccccctttcgccagctgg<br>cgtaatagcgaagaggcccgcaccgatcgcccttcccaacagttgcgcagcctgaatggcgaatggcgcgacgcgccctgtagcggcgcatta<br>agcgcggcgggtgtggtggttacgcgcagcgtgaccgctacacttgccagcgccctagcgcccgctcctttcgctttcttcccttcctttctcgcca<br>cgttcgccggctttccccgtcaagctctaaatcgggggctccctttagggttccgatttagtgctttacggcacctcgaccccaaaaaacttgattag<br>ggtgatggttcacgtagtgggccatcgccctgatagacggtttttcgccctttgacgttggagtccacgttctttaatagtggactcttgttccaaactg<br>gaacaacactcaaccctatctcggtctattcttttgatttataagggattttgccgatttcggcctattggttaaaaaatgagctgatttaacaaaaatttaa<br>cgcgaattttaacaaaatattaacgtttacaatttcctgatgcggtattttctccttacgcatctgtgcggtatttcacaccgcatagggtaataactgatat<br>aattaaattgaagctctaatttgtgagtttagtatacatgcatttacttataatacagtttttagttttgctggccgcatcttctcaaatatgcttcccagcct<br>gcttttctgtaacgttcaccctctaccttagcatcccttccctttgcaaatagtcctcttccaacaataataatgtcagatcctgtagagaccacatcatcc<br>acggttctatactgttgacccaatgcgtctcccttgtcatctaaacccacaccgggtgtcataatcaaccaatcgtaaccttcatctcttccacccatgt<br>ctctttgagcaataaagccgataacaaaatctttgtcgctcttcgcaatgtcaacagtacccttagtatattctccagtagatagggagcccttgcatga<br>caattctgctaacatcaaaaggcctctaggttcctttgttacttcttctgccgcctgcttcaaaccgctaacaatacctgggcccaccacaccgtgtgc<br>attcgtaatgtctgcccattctgctattctgtatacacccgcagagtactgcaatttgactgtattaccaatgtcagcaaattttctgtcttcgaagagtaa<br>aaaattgtacttggcggataatgcctttagcggcttaactgtgccctccatggaaaaatcagtcaagatatccacatgtttttagtaaacaaattttgg<br>gacctaatgcttcaactaactccagtaattccttggtggtacgaacatccaatgaagcacacaagtttgtttgcttttcgtgcatgatattaaatagcttg<br>gcagcaacaggactaggatgagtagcagcacgttccttatatgtagctttcgacatgatttatcttcgtttcctgcaggtttttgttctgtgcagttgggtt<br>aagaatactgggcaatttcatgtttcttcaacactacatatgcgtatatataccaatctaagtctgtgctccttccttcgttcttccttctgttcggagattac<br>cgaatcaaaaaaaatttcaaagaaaccgaaatcaaaaaaaagaataaaaaaaaaaatgatgaattgaattgaaaagctgtggtatggtgcactctcagt

	acaatctgctctgatgccgcatagttaagccagccccgacacccgccaacacccgctgacgcgccctgacgggcttgtctgctcccggcatccgcttacagacaagctgtgaccgtctccgggagctgcatgtgtcagaggttttcaccgtcatcaccgaaacgcgcga
Super piggyBac transposase	atgggttcctccttggatgatgaacatattttgtctgccttgttgcaatccgatgatgaattggttggtgaagattccgattccgaagtttctgatcatgtttccgaagatgatgttcaatctgatactgaagaagccttcatcgacgaagttcatgaagttcaacctacatcttccggttccgaaattttggatgaacaaaacgttatcgaacagccaggttcttcattggcttctaacagaattttgaccttgccacaaagaaccatcaggggtaaaaacaaacattgctggtctacttctaagtccaccagaagatcaagagtttctgctttgaacatcgtcagatctcaaaggggtccaactagaatgtgtagaaatatctacgatcccctgttgtgcttcaagttgtttttaccgacgaaatcatctccgaaatcgttaagtggactaacgccgaaatttccttgaagagaagagaatctatgacctctgctactttcagagatacaaacgaagatgagatctacgccttcttcggtattttggttatgactgctgttagaaaggacaaccatatgtctaccgatgatttgttcgacaggtctttgtctatggtttacgtgtctgttatgtccagagatagattcgacttcttgatcagatgcttgagaatggatgacaagtctattagaccaaccttgagagaaaacgatgttttcactccagttagaaagatctgggacttgttcattcatcaatgcatccaaaactataccccaggtgctcatttgactattgatgaacaactgttgggtttcagaggtagatgtcctttcagagtttacattccaaacaagccatctaagtacggcatcaagattttgatgatgtgtgactctggtacgaagtacatgattaacggtatgccatacttaggtagaggtactcaaactaatggtgttccattgggtgagtactacgtcaaagaattgtctaaaccagttcacggttcctgcagaaacattacttgtgataattggttcacctccattccattggctaagaatttgttgcaagagccatacaagttgaccatcgttggtactgttagatccaacaagagagaaatcccagaagtcttgaagaacagtagatcaagaccagttggtacttctatgttctgttttgatggtccattgaccttggtttcttacaaacctaaaccagccaagatggtgtacttgttgtcatcttgtgatgaagatgcctccattaacgaatctactggtaaaccacaaatggtcatgtactacaatcagaccaaaggtggtgttgataccttggatcaaatgtgttctgttatgacctgttccagaaagactaatagatggccaatggctttgttgtacggcatgatcaatattgcctgtatcaactccttcatcatctactcccataacgtttcttctaagggtgaaaaggtccagtccagaaaaaagtttatgaggaacctgtatatgtccctgacctcttcattcatgagaaagagattggaagctccaactttgaagagatacttgagagacaacatctccaacatcttgcctaaagaagttccaggtacatctgatgattcaaccgaagaaccagttatgaagaagagaacttactgtacctactgtccctccaaaattagacgtaaagctaacgcttcttgtaagaagtgcaagaaggttatctgcagagaacacaacattgacatgtgtcaatcctgcttctag
glyceraldehyde-3-phosphate dehydrogenase (GAP) promoter	tcattatcaatactcgccatttcaaagaatacgtaaataattaatagtagtgattttcctaactttatttagtcaaaaaattagccttttaattctgctgtaacccgtacatgcccaaaataggggggcgggttacacagaatatataacatcgtaggtgtctgggtgaacagtttattcctggcatccactaaatataatggagcccgctttttaagctggcatccagaaaaaaaaaagaatcccagcaccaaaatattgttttcttcaccaaccatcagttcataggtccattctcttagcgcaactacagagaacaggggcacaaacaggcaaaaaacgggcacaacctcaatggagtgatgcaacctgcctggagtaaatgatgacacaaggcaattgacccacgcatgtatctatctcattttcttacaccttctattaccttctgctctctctgatttggaaaaagctgaaaaaaaaggttgaaaccagttccctgaaattattcccctacttgactaataagtatataaagacggtaggtattgattgtaattctgtaaatctatttcttaaacttcttaaattctacttttatagttagtctttttttttagttttaaaacaccagaacttagtttcga
CYC1 terminator sequence	tcatgtaattagttatgtcacgcttacattcacgccctccccccacatccgctctaaccgaaaaggaaggagttagacaacctgaagtctaggtccctatttattttttttatagttatgttagtattaagaacgttatttatatttcaaatttttcttttttttctgtacagacgcgtgtacgcatgtaacattatactgaaaaccttgcttgagaaggttttgggacgctcgaaggctttaatttgc
SNR52 terminator	tttatttttgtcactattgttatgtaaaatgccacctctgacagtatggaacgcaaacttctgtctagtggataacagaatttttctatggccaattta

Native leucine promoter	aactgtgggaatactcaggtatcgtaagatgcaagagttcgaatctcttagcaaccattatttttttcctcaacataacgagaacacacaggggcgctatcgcacagaatcaaattcgatgactggaaatttttttgttaatttcagaggtcgcctgacgcatatacctttttcaactgaaaaattgggagaaaaaggaaaggtgagaggccggaaccggcttttcatatagaatagagaagcgttcatgactaaatgcttgcatcacaatacttgaagttgacaatattatttaaggacctattgttttttccaataggtggttagcaatcgtcttactttctaacttttcttaccttttacatttcagcaatatatatatatatttcaaggatataccattcta
Clo051	EGIKSNISLLKDELRGQISHISHEYLSLIDLAFDSKQNRLFEMKVLELLVNEYGFKGRHLGGSRKPDGIVYSTTLEDNFGIIVDTKAYSEGYSLPISQADEMERYVRENSNRDEEVNPNKWWENFSEEVKKYYFVFISGSFKGKFEEQLRRLSMTTGVNGSAVNVVNLLLGAEKIRSGEMTIEELERAMFNNSEFILKY
URA3 left gRNA	cttgtcatctaaacccacac
URA3 right gRNA	gtatagaaccgtggatgatg
LEU2 left gRNA	gatatcaccaaacatgttgc
LEU2 right gRNA	tccgttatcccaggttcctt
ADH1 promoter	acatgtaggtggcggaggggagatatacaatagaacagataccagacaagacataatgggctaaacaagactacaccaattacactgcctcattgatggtggtacataacgaactaatactgtagccctagacttgatagccatcatcatatcgaagtttcactaccctttttccatttgccatctattgaagtaataataggcgcatgcaacttcttttcttttttttttcttttctctctcccccgttgttgtctcaccatatccgcaatgacaaaaaaatgatggaagacactaaaggaaaaaattaacgacaaagacagcaccaacagatgtcgttgttccagagctgatgaggggtatctcgaagcacacgaaactttttccttccttcattcacgcacactactctctaatgagcaacggtatacggccttccttccagttacttgaatttgaaataaaaaaaagtttgctgtcttgctatcaagtataaatagacctgcaattattaatcttttgtttcctcgtcattgttctcgttccctttcttccttgtttctttttctgcacaatatttcaagctataccaagcatacaatcaactatctcatataca

Table S1. Sequences of the indicated features are provided.
